# Oral polio vaccine response in the MAL-ED birth cohort study: Considerations for polio eradication strategies

**DOI:** 10.1016/j.vaccine.2018.05.080

**Published:** 2019-01-07

**Authors:** William K. Pan, Jessica C. Seidman, Asad Ali, Christel Hoest, Carl Mason, Dinesh Mondal, Stacey L. Knobler, Pascal Bessong, The MAL-ED Network Investigators

**Affiliations:** a Duke Global Health Institute, Duke University, Trent Hall, 310 Trent Drive, Durham, NC 27710, USA; b Fogarty International Center/National Institutes of Health, Bethesda, MD, USA; c Aga Khan University, Karachi, Pakistan; d Armed Forces Research Institute of Medical Sciences, Bangkok, Thailand; e ICDDR, B, Dhaka, Bangladesh; f University of Venda, Thohoyandou, South Africa

**Keywords:** Oral polio vaccination, Poliomyelitis, Enteropathogen infection, Home environment, MAL-ED, The Etiology, Risk Factors and Interactions of Enteric Infections and Malnutrition and the Consequences for Child Health and Development, EPI, Expanded Program on Immunization, BGD, Dhaka, Bangladesh, BRF, Fortaleza, Brazil, INV, Vellore, India, NEB, Bhaktapur, Nepal, PEL, Loreto, Peru, PKN, Naushero Feroze, Pakistan, SAV, Venda, South Africa, TZH, Haydom, Tanzania, OPV, Oral Poliovirus Vaccine, IPV, inactivated oral poliovirus vaccine, cVDPV, circulating vaccine-derived poliomyelitis, tOPV, trivalent Oral Poliovirus Vaccine, WAMI, Water/sanitation, household Assets, Maternal education, and household Income index, GMT, geometric mean titer

## Abstract

**Background:**

Immunization programs have leveraged decades of research to maximize oral polio vaccine (OPV) response. Moving toward global poliovirus eradication, the WHO recommended phased OPV-to-IPV replacement on schedules in 2012. Using the MAL-ED prospective birth cohort data, we evaluated the influence of early life exposures impacting OPV immunization by measuring OPV response for serotypes 1 and 3.

**Methods:**

Polio neutralizing antibody assays were conducted at 7 and 15 months of age for serotypes 1 and 3. Analyses were conducted on children receiving ≥3 OPV doses (n = 1449). History of vaccination, feeding patterns, physical growth, home environment, diarrhea, enteropathogen detection, and gut inflammation were examined as risk factors for non-response [Log_2_(titer) < 3] and Log_2_(titer) by serotype using multivariate regression.

**Findings:**

Serotype 1 seroconversion was significantly higher than serotype 3 (96.6% vs. 89.6%, 15 months). Model results indicate serotypes 1 and 3 failure was minimized following four and six OPV doses, respectively; however, enteropathogen detection and poor socioeconomic conditions attenuated response in both serotypes. At three months of age, bacterial detection in stool reduced serotype 1 and 3 Log_2_ titers by 0.34 (95% CI 0.14–0.54) and 0.53 (95% CI 0.29–0.77), respectively, and increased odds of serotype 3 failure by 3.0 (95% CI 1.6–5.8). Our socioeconomic index, consisting of Water, Assets, Maternal education, and Income (WAMI), was associated with a 0.79 (95% CI 0.15–1.43) and 1.23 (95% CI 0.34–2.12) higher serotype 1 and 3 Log_2_ titer, respectively, and a 0.04 (95% CI 0.002–0.40) lower odds of serotype 3 failure. Introduction of solids, transferrin receptor, and underweight were differentially associated with serotype response. Other factors, including diarrheal frequency and breastfeeding practices, were not associated with OPV response.

**Interpretation:**

Under real-world conditions, improved vaccination coverage and socio-environmental conditions, and reducing early life bacterial exposures are key to improving OPV response and should inform polio eradication strategies.

## 1 Background

Poliomyelitis, caused by infection with poliovirus serotypes 1, 2, or 3, can cause severe paralysis and death. Humans are the only known host for poliovirus and transmission is primarily fecal-oral. Two vaccines can prevent polio: (live attenuated) oral poliovirus vaccine (OPV) and parenteral inactivated poliovirus vaccine (IPV). Until 2016, OPV was the primary vaccine recommended by the World Health Organization (WHO) and its use contributed significantly to the near elimination of paralytic polio. Currently, wild poliovirus (WPV) is endemic in just Nigeria, Pakistan and Afghanistan. Global eradication of WPV serotype 2 was certified in 2015 and the last reported WPV serotype 3 case in 2012 leaving serotype 1 as the primary concern [1], [2].

Global eradication of poliomyelitis is feasible. Confirmed WPV cases fell from 416 in 2013 to 22 in 2017, with just 8 cases as of May 2018 [1]. Circulating vaccine-derived poliomyelitis (cVDPV) cases have also declined, from 66 cases in 2013 (65 due to cVDPV type 2) to 5 in 2016 (three type 1 and two type 2). Although cVDPV cases spiked in 2017 (96 cases, all cVDPV type 2), only four cases (all type 2) have been identified through May 2018 [3]. These declines follow WHO recommendations to modify routine immunization strategies by: including at least one IPV dose; shifting from trivalent OPV (tOPV) to monovalent or bivalent OPV serotypes 1 and 3 with IPV; and eventual OPV cessation to eliminate cVDPV [4], [5]. Despite improved serum response with IPV [6], [7], [8], some question its effectiveness against transmission in regions with poor sanitation due to limited induction of mucosal immunity [9], [10]. Strong humoral and secretory immunity are needed to prevent poliomyelitis and poliovirus transmission.

The Etiology, Risk Factors and Interactions of Enteric Infections and Malnutrition and the Consequences for Child Health and Development (MAL-ED) prospective birth cohort study was initiated in eight diverse low and middle-income populations [Dhaka, Bangladesh (BGD); Fortaleza, Brazil (BRF); Vellore, India (INV); Bhaktapur, Nepal (NEB); Loreto, Peru (PEL); Naushero Feroze, Pakistan (PKN); Venda, South Africa (SAV); and Haydom, Tanzania (TZH)] to evaluate the impact of enteropathogen infection and undernutrition on child development, growth, and vaccine response [11]. MAL-ED sites employ varying polio vaccination schedules [12]; all sites administered tOPV and one site (SAV) administered tOPV with IPV. MAL-ED offers a unique opportunity to evaluate OPV programs at a time when their effectiveness is believed to have peaked.

We evaluated factors influencing OPV response following at least three doses in MAL-ED sites exclusively using tOPV between 2009 and 2013. Polio vaccine response has been extensively studied since the 1950s, including antibody inhibition (i.e., serotype 2 placental antibody interference with other serotypes, and/or breastfeeding intensity) [13], [14], [15], [16], [17], [18], [19], [20], OPV administration timing and formulation (i.e., higher quantities of serotype3 in tOPV) [21], [22], [23], [24], and nutritional status (e.g., [25], [26]). Past research has highlighted the relationship between diarrhea and OPV response [27], [28], while recent studies focused on the nutrition-diarrhea cycle and associated environmental enteropathy impacting OPV response [29], [30], [31]. Suboptimal seroconversion rates following three-dose tOPV regimens have been observed in many low-income countries [20], [32], [33]. We aimed to identify factors across multiple contexts contributing to reduced OPV response at the apex of OPV global use. We focus on the following issues believed to impact OPV response: (1) variations in vaccine timing; (2) enteric infection, diarrhea and malnutrition; and (3) socioeconomic status and quality of the home environment.

## 2 Methods

### 2.1 Study design and participants

The MAL-ED study, described elsewhere [11], [34], differs from much of the polio vaccine response literature that primarily describes controlled, clinical trials; in contrast, MAL-ED was an observational study that evaluated vaccine response under real-world conditions, which include supplemental immunization to maximize OPV response. MAL-ED enrolled participants within 17 days of birth and followed them intensively for the first two years of life. Children were included in this analysis if they received at least three doses of OPV before the protocol blood draws; those receiving IPV were excluded. The study was conducted under human use research protocols approved by local and/or national ethical review committees at each site. Signed consent was obtained for participation.

### 2.2 Assessment of OPV response

Blood collection was scheduled at 7 and 15 months of age ±14 days to accommodate participant availability and illness. Poliovirus serum neutralizing antibody titers were measured using WHO-standardized microneutralization assays [12], [35]. The primary outcomes were serotype-specific non-response, defined as Log_2_ (titer) < 3 (hereafter called seroconversion failure), and Log_2_(titer). Exposures of interest are briefly defined below and in Supplemental Table 1.

### 2.3 Vaccination history

Children were vaccinated at local health facilities and during vaccine campaigns; not by the MAL-ED study. Structured monthly questionnaires were administered to record dates of vaccination, along with a quarterly assessment of confirmed dates and receipt of vaccination [12], [36]. Locally-defined rainy seasons were also identified to classify OPV timing.

### 2.4 Enteropathogen detection, diarrhea, and nutrient status

Twice-weekly household surveillance captured the occurrence of diarrheal symptoms (≥3 loose stools in 24 h) [34]. Diarrheal stools collected during household visits and non-diarrheal stools (separated by ≥2 diarrhea-free days) collected monthly in the first year and quarterly in the second year, were tested for ≥40 enteropathogens [37]. Frequency of diarrhea episodes and enteropathogen detection scores were computed at early ages (4, 8, 12 and 16 weeks) and at the time of blood draw (7 and 15 months). Diarrhea frequency was additionally assessed 1, 3, and 5 days before and after an OPV dose. Enteropathogen scores were computed as the cumulative number of pathogen detections divided by the total stools collected up to a specified age. Scores were computed separately by stool type (diarrhea vs. non) and for all stools combined. We evaluated scores for individual ((*Campylobacter*, *Cryptosporidium*, enteroaggregative *Escherichia coli* (EAEC), *Giardia*) and pathogen categories (bacteria, viruses, parasites, all combined). Finally, gut inflammation and permeability were measured using fecal α-1 antitrypsin, neopterin, myeloperoxidase, and urinary lactulose:mannitol ratio [38].

Nutritional status was measured using monthly anthropometry and serum biomarkers at 7 and 15 months. Monthly anthropometry (length (cm), weight (kg)) was converted to length-for-age (LAZ), weight-for-age (WAZ), weight-for-length (WFL) Z-scores and categorized (stunted LAZ < −2, wasted WAZ < −2, underweight WFL < −2) based on WHO standards [39]; quality control procedures revealed bias in length measures from Naushero Feroze (Pakistan) thus children from this site were excluded in analyses involving length. Growth velocity during the first three months of life was also computed. Biomarkers of nutrient status (retinol, ferritin, transferrin receptor, hemoglobin, zinc, alpha-1-acid glycoprotein) were measured from the same blood samples as OPV titers [40].

Infant feeding patterns, including, frequency of breastfeeding and age at introduction of non-breastmilk liquids and solids, were recorded during household surveillance visits [40]. Breastfeeding status was characterized as exclusive, partial, or predominant (Supplemental Table 1 for definitions).

### 2.5 Socioeconomic status and HOME environment

A socioeconomic status index was developed for the MAL-ED study [41]. The index is a composite of: Water/sanitation, household Assets, Maternal education, and household Income (WAMI, components range from 0 to 8; components are summed and divided by 32; WAMI ranges from 0 to 1). WAMI components were measured at 6, 12 and 18 months; however, little variation existed over time, so mean scores for these time points were used.

A modified version of the Home Observation for the Measurement of the Environment (HOME [42], [43]) was administered by the MAL-ED study at 6, 24, and 36 months of age [44]. Two HOME factor scores (range 0–4) were computed: Clean and Safe Environment, which reflects (permanent) environments conducive to the safety and health of the child; and Child Cleanliness, which reflects cleanliness of the child [45]. This analysis uses the 6 month scores and change from 6 to 24 months.

### 2.6 Statistical analysis

Analyses focused on response to serotypes 1 and 3. Univariate analyses were used to compare characteristics across sites and to assess differences in response (seroconversion failure and Log_2_ titers) across factors using the Cochrane-Armitage Trend test (for continuous factors divided into ordered categories) and t-tests (for factors consisting of two groups). Two multivariate models were fit for serotypes 1 and 3 with random effects to adjust for correlation within site and a fixed effect for age at sample collection (in months): a logistic model for serotype failure and linear mixed model for Log2 titer (See supplementary material). Model selection was guided using AIC statistics. Significance was interpreted at the 0.05 level; however, with five thematic areas evaluated (vaccination history, infant feeding practices, nutritional status, enteric infection, home environment), a Bonferonni corrected alpha-level of 0.01 is also provided. All analyses were conducted using SAS 9.4 (SAS Institute Inc., Cary, NC, USA) [46].

### 2.7 Role of the funding sources

The Bill & Melinda Gates Foundation did not play any role in the writing of the manuscript nor in the study design, data collection, data analysis, or interpretation of results. The corresponding author had full access to all the data in the study and final responsibility for the decision to submit for publication.

## 3 Results

**Fig. 1:**
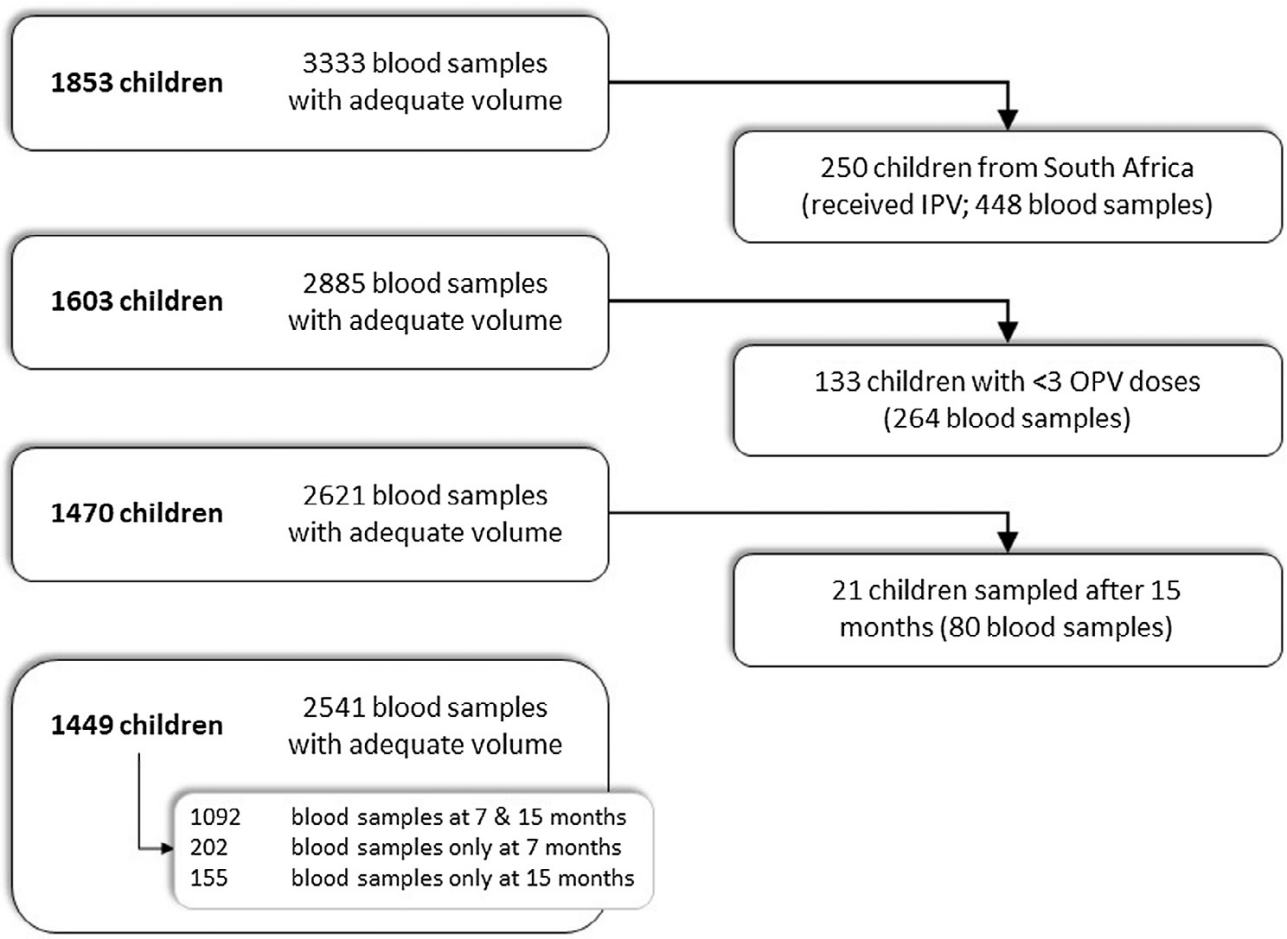
**Child samples selected for OPV response study.** Blood sample collection from each child was scheduled at seven and 15 months of age. All blood samples with sufficient volume were tested for poliovirus serotype 1, 2, and 3 serum neutralizing antibody assays. Children administered inactivated poliovirus vaccines were excluded, all of whom were from the MAL-ED cohort study site in South Africa. Blood samples collected from children receiving less than three documented doses of oral poliovirus vaccine prior to the date of blood collection were also excluded. The 2541 samples included in this analysis represent 1449 children from seven MAL-ED cohort study sites. 1092 of 1449 children (75%) contributed samples at both time points.

In total, 1862 children provided blood samples and poliovirus titers for serotypes 1–3 were obtained from 1853 children (Fig. 1). South Africa uses a combined OPV-IPV schedule, thus 250 children from Venda received IPV and were excluded from this analysis. Vaccine schedules were comparable across the remaining sites (Table 1), with all children scheduled to receive a minimum of three OPV doses by six months of age [47]. Among the eligible 1603 children, 224 (14.0%) and 118 (7.4%) did not receive three OPV doses by seven and 15 months of age, respectively (Table 1). However, due to some delayed blood draws, 133 children were excluded from this analysis who had not received sufficient OPV doses prior to actual time of sampling.

**Table 1:** OPV schedule and percent of children enrolled who received at least 3 OPV doses by 7, 15, and 24 months of age by site. Schedules indicating monthly doses are converted to weeks by multiplying by 4. e.g., Bangladesh has a 4th dose at 9 months (36 weeks); India has a 5th dose between 16 and 24 months; Brazil and Peru have doses at 2, 4 and 6 months (8, 16, 24 weeks), with Brazil administering another dose at 15 months (60 weeks); and Tanzania administered OPV at 1, 2, and 3 months (4, 8, 12 weeks).

Site	N^a^	OPV schedule (weeks)	Percent (%) of all children enrolled who received ≥3 doses by age^b^		
			7 months	15 months	24 months
Dhaka, Bangladesh (BGD)	223	6, 10, 14, 36	95.5	97.8	98.7
Bhaktapur, Nepal (NEB)	232	6, 10, 14	100	100	100
Vellore, India (INV)	233	0, 6, 10, 14, 64–96	92.7	97.0	100
Naushero Feroze, Pakistan (PKN)	260	0, 6, 10, 14	100	100	100
Fortaleza, Brazil (BRF)	186	8, 16, 24, 60	61.3	87.1	90.3
Loreto, Peru (PEL)	259	8, 16, 24	83.0	95.4	95.8
Haydom, Tanzania (TZH)	210	0, 4, 8, 12	61.4	66.7	67.6

Total	1603		86.0	92.6	93.8

a Sample sizes include all children enrolled in the study who provided a blood sample.

b Total doses are computed to the exact monthly age and not the age of the blood draw; children with fewer than three doses at the time of the blood draw were excluded from analysis.

Seroconversion rates (and geometric mean titers [GMT]) for serotypes 1 and 3 were 1227/1294 (95%; 9.47) and 1130/1294 (87%; 8.30), respectively at 7 months, and 1205/1247 (97%; 9.39) and 1118/1247 (90%; 8.15) at 15 months (Fig. 2a, Fig. 2b). Titers were highest in Fortaleza and Bhaktapur (Fig. 2a, Fig. 2b); however, titer distributions varied: at 15 months, serotype 1 seroconversion ranged from 153/172–131/132 (89–99%), and for serotype 3, response ranged from 46/64–128/132 (72–97% in Haydom and Fortaleza).

**Fig. 2a:**
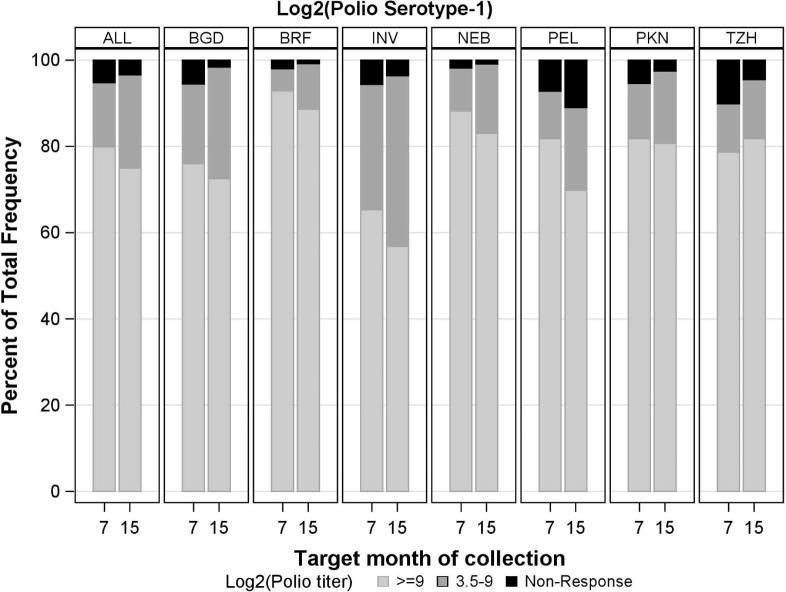
**Poliovirus serotype 1 neutralization titer distributions by site and scheduled month of blood collection.** Site locations; Dhaka, Bangladesh (BGD), Foratleza, Brazil (BRF); Vellore, India (INV); Bhaktapur, Nepal (NEB); Loreto, Peru (PEL); Naushero Feroze, Pakistan (PKN); Haydom, Tanzania (TZH).

**Fig. 2b:**
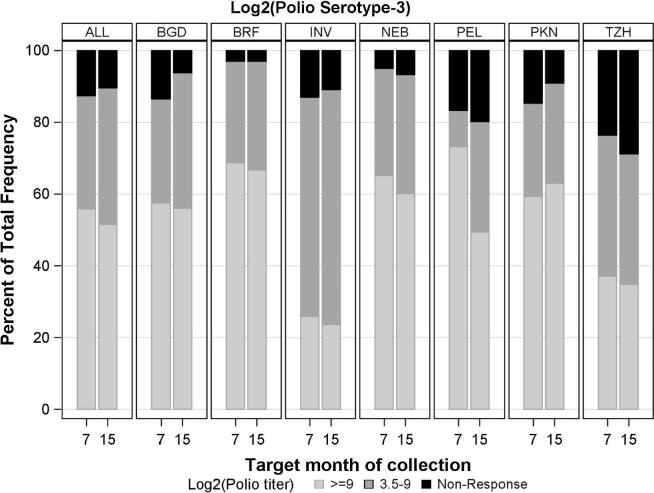
**Poliovirus serotype 3 neutralization titer distributions by site and scheduled month of blood collection.** Site locations; Dhaka, Bangladesh (BGD), Foratleza, Brazil (BRF); Vellore, India (INV); Bhaktapur, Nepal (NEB); Loreto, Peru (PEL); Naushero Feroze, Pakistan (PKN); Haydom, Tanzania (TZH).

Several factors were identified as influencing OPV response in univariate and multivariate analyses; these are presented by groups (vaccine history, enteropathy and nutrition, home environment). Model results (Table 2a, Table 2b) are described by group (aspects of model fit are presented in Supplemental). Importantly, children in Loreto and Vellore had the highest adjusted odds of failure and the lowest predicted serotype 1 Log_2_ titers. In contrast, children in Fortaleza and Bhaktapur had the lowest odds of failure, with children in Fortaleza and Haydom having the highest titers. For serotype 3, children in Loreto and Haydom had the highest adjusted odds of failure, and children in Vellore had significantly lower titers compared to all other sites (p < 0.0001). Children in Fortaleza had significantly lower odds of serotype 3 failure compared to children in Vellore, Bhaktapur, Loreto and Haydom and significantly higher titers than children in Dhaka, Vellore, Bhaktapur and Haydom. Tables 2a and 2b have very poor alignment in the PDF Proof - the 95% CI wraps into a second line and it makes it very hard to read the tables. Can this be corrected?

**Table 2a:** Final multivariate models for serotype 1 failure and serotype 1 Log_2_ titer among children receiving a minimum of 3 OPV doses. Variables with no estimate indicated were not part of the final multivariate model (e.g., diarrheal bacterial score at 3 months was not part of the final model for serotype 1 failure). Effect sizes for these and other covariates are in Supplemental.

		Serotype 1 failure^2^^,^^3^		Serotype 1 Log_2_ titer^2^	
		Odds ratio	(95% CI)	Beta	(95% CI)
**Site** (REF = BRF)	BGD	5.6	(0.49–63.5)	−0.46†	(−0.89 to 0.03)
	INV	7.8*	(0.69–87.1)	−0.92†††	(−1.39 to 0.46)
	NEB	1.5	(0.15–16.2)	−0.24	(−0.63 to 0.15)
	PEL	15.2†	(1.55–149)	−0.46†	(−0.9 to 0.02)
	PKN	6.6	(0.47–92.8)	−0.13	(−0.58 to 0.33)
	TZH	4.8	(0.38–59.6)	0.14	(−0.49 to 0.76)

**Age at blood draw** (in months)^1^		1.02	(0.92–1.10)	−0.03††	(−0.04 to 0.01)

**OPV doses** (REF = 3)	4	0.24†	(0.08–0.70)	0.33††	(0.11–0.56)
	5+	0.07†††	(0.02–0.30)	0.55†††	(0.31–0.8)

**Enteropathogen scores, diarrheal stools**					
All pathogens	At blood draw	1.89††	(1.23–2.90)		
Bacteria	at 3 months			−0.34†††	(−0.54 to 0.14)

**Enteropathogen scores, diarrheal & non-diarrheal stools**					
Bacteria	At blood draw			−0.33††	(−0.57 to 0.08)

**Nutritional variables**					
Age solids introduced (REF ≥ 4 m)	2–4 m	4.2††	(1.59–11.0)	−0.29††	(−0.5 to 0.07)
	<2 m	2.5	(0.83–7.50)	−0.26†	(−0.52 to 0)
Plasma transferritin receptor (μg/mL, REF ≥ 8.9)	2.9–8.3			−0.16*	(−0.32 to 0.01)
	<2.9			−0.33†	(−0.6 to 0.06)

**Socioeconomic status & home environment**					
WAMI, assets component^1^		0.83	(0.66–1.05)		
WAMI (overall index)^1^				0.79†	(0.15–1.43)
Child cleanliness (6–24 m change, REF: no change)^1^	Worse	3.4†	(1.16–10.2)		
	Better	3.1	(0.8–12.0)		
Age * child cleanliness (6–24 m change)	Worse	0.95	(0.82–1.10)		
	Better	0.66†	(0.45–0.90)		

††† p < 0.001.

†† p < 0.01.

† p < 0.05.

* p < 0.10.

1 Age was centered at 7 months, WAMI (range 0–1) was centered at 0.5, WAMI-Assets (range 0–8) was centered at 4, and change in Child Cleanliness ranges from −4 to 4.

2 The intercept for Serotype 1 failure was −6.448; the intercept for Log2 (Serotype 1 titer) is 9.73. The serotype 1 failure model used a final sample size of 2316 non-missing observations; the serotype 1 titer model used 2378. Missing data were primarily due to missing enteropathogen data at 3 months, plasma transferritin receptor, and HOME environment measures.

3 For seroconversion failure, we can replace the diarrhea-associated pathogen score at the blood draw with the diarrhea-associated bacteria score at the blood draw to provide approximately the same model, with a slightly higher AIC. The odds ratio for a one-unit change in the diarrheal bacteria score was 1.84, with a p-value of 0.0254. The correlation between the diarrheal pathogen and bacteria score was high (rho = 0.90), indicating that both variables could not be included in the model.

**Table 2b:** Multivariate model results for serotype 3 failure and serotype 3 Log_2_ titer among children receiving a minimum of 3 OPV doses. Variables with no estimate indicated were not part of the final multivariate model. Effect sizes for these and other covariates are in Supplemental.

		Serotype 3 failure^2^^,^^4^		Serotype 3 Log_2_ titer^2^	
		OR	(95% CI)	Beta	(95% CI)
**Site** (REF = BRF)	BGD	7.12*	(0.96–52.6)	−0.58†	(−1.16 to 0)
	INV	13.2*	(1.61–108.3)	−1.76†††	(−2.39 to 1.14)
	NEB	7.45*	(1.06–52.3)	−0.56†	(−1.08 to 0.04)
	PEL	16.6††	(2.26–122.4)	−0.42	(−1.01 to 0.16)
	PKN	8.59*	(0.99–74.8)	−0.41	(−1.02 to 0.21)
	TZH	16.1†	(1.45–178.8)	−1.08†	(−1.93 to 0.24)

**Age at blood draw** (in months)^1^		0.98	(0.93–1.04)	−0.06†††	(−0.08 to 0.03)

**OPV doses** (REF = 3)	4	0.37†	(0.17–0.81)	0.42††	(0.14–0.7)
	5+	0.11†††	(0.04–0.28)	0.85†††	(0.54–1.15)

**Enteropathogen scores, non-diarrheal stools**					
Parasites	At blood draw			−0.41	(−0.77 to 0.04)

**Enteropathogen scores, diarrhea & non-diarrhea stools**					
Bacteria	At 3 months	3.01†††	(1.56–5.78)	−0.53†††	(−0.77 to 0.29)
Parasites	At blood draw	7.56†	(1.51–37.95)		

**Nutritional variables**					
% days underweight (REF ≥ 50%)^3^	10–49%	0.34†	(0.12–0.93)	0.3	(−0.08 to 0.69)
	<10%	0.27††	(0.1–0.68)	0.41†	(0.05–0.77)
Plasma transferretin receptor (μg/mL, REF ≥ 8.9)	2.9–8.3			−0.18*	(−0.39 to 0.02)
	<2.9			−0.41†	(−0.74 to 0.07)

**Socioeconomic status & home environment**					
WAMI (overall index)^1^		0.04†	(0–0.47)	1.23††	(0.34–2.12)

††† p < 0.001.

†† p < 0.01.

† p < 0.05.

* p < 0.10.

1 Age was centered at 7 months, WAMI is centered at 0.5 (range 0–1).

2 The intercept for Serotype 3 failure was −5.9861; the intercept for Log2 (Serotype 3 titer) is 8.54. The sample size for the serotype 3 failure model is 2302, for the Log2(Serotype 3) model 2378.

3 10% or fewer days underweight is approximately equal to one month of seven months measured as underweight at the 7 m blood draw compared to 50% corresponding to approximately four of seven months.

4 Replacing WAMI with WAMI-Sanitation (range 0–8) results in an identical model fit. A one-unit change in WAMI Sanitation resulted in a 0.74 (95% CI 0.60–0.92, p = 0.0073) lower odds of serotype 3 failure. In addition, adding Urinary Mannitol z-score at 6 months of age provided slightly improved fit to the original model (with both WAMI and WAMI-Sanitation); however, it resulted in a loss of 200 observations (∼9% of the data). This second model did not alter odds ratio estimates for any covariates listed above for the final serotype 3 failure model and indicated that a one-unit increase in the 6-months Mannitol z-score would reduce the odds of serotype 3 failure by 0.67 (95% CI 0.47–0.94, p = 0.0227).

### 3.1 Vaccine history

By 15 months of age, 99/1603 enrolled children (6.2%) had not received three OPV doses (Table 1). Children in Naushero Feroze received the highest mean number of OPV doses (8.2 by 12 months), while children in Loreto and Fortaleza received the fewest (3.0 and 3.7, respectively). Among the three sites with a birth dose, delays were observed where the average age of the first dose was 14 (Vellore), 16 (Naushero Feroze), and 40 (Haydom) days.

OPV response was strongly associated with the number of doses received in univariate analyses (Table 3a). Children receiving more OPV doses had significantly improved responses for serotypes 1 and 3 at 7 and 15 months; however, the effect was attenuated if doses were administered during the rainy season. Children with a delayed first dose of OPV had poorer response to each serotype (Table 3a).

**Table 3a:** Vaccine history & OPV response.

Results at 7 months followed the same trends as those observed at 15 months.

^†^ p < 0.05 from Cochrane-Armitage Trend test.

^††^ p < 0.01 from Cochrane-Armitage Trend test.

^†††^ p < 0.0001 from Cochrane-Armitage Trend test.

**Table 3b:** Enteropathogens, diarrhea and nutrient status compared to OPV response.

Results at 7 months followed the same trends as those observed at 15 months.

^1^ Cutpoints used represent tertiles for the specific variable. For example, <10, 10–50, and >50 days are tertiles for diarrhoea frequency up to the 15 month blood draw.

^2^ Growth velocity tertiles were computed as the difference in length at 3 months and the earliest measure of length divided by the difference in days between the two measures. Tertiles were 0–0.105 (T1), 0.105–0.124 (T2); and >0.124 (T3).

^†^ p < 0.05 from Cochrane-Armitage Trend test.

^††^ p < 0.01 from Cochrane-Armitage Trend test.

^†††^ p < 0.0001 from Cochrane-Armitage Trend test. Same general comments as Table3a, particularly the alignment of the categories and the notches in the box plot. The PDFproof has the alignment even further off.

Serotype 1 response was strongly associated with the number of OPV doses received in the multivariate model; however, improvements were minimal above 5 doses (Table 2a). When comparing children who received 3, 4, 5, 6 and 7+ doses, there were no significant differences in failure, except a borderline significant difference for 4 vs. 6 doses (OR = 1.37, 95%CI −0.09 to 2.83). Similarly, in the serotype Log_2_ 1 titer model no differences were observed for receiving 5, 6 or 7+ doses; however, a higher titer was achieved for 4 vs. 3 doses (0.41, 95% CI 0.19–0.64) and 5 vs 4 doses (0.24, 95% CI 0.013–0.466).

Serotype 3 responses improved with more OPV doses in the multivariate model (Table 2b). Odds of serotype 3 failure declined up to 7+ OPV doses, and Log_2_ titers significantly improved up to 5 doses. Only children in Naushero Feroze received 8+ doses; therefore, a second serotype 3 evaluation was conducted excluding Naushero Feroze: no change was indicated in the Log_2_ model; however, odds of failure did not significantly improve beyond 5 doses; therefore, the final multivariate model reports effects up to 5 or more OPV doses (Table 2b) .

### 3.2 Enteropathogen detection, diarrhea, and nutritional status

Enteropathogen detection and nutritional status varied by site (Table 4), as previously reported [48], [49], [50]. Enteropathogen burden was highest in Naushero Feroze and Haydom (Table 4), and the average total days of diarrhea at 7 m ranged from 0.3 (Fortaleza) to 29.2 (Naushero Feroze). All sites reported at least 20% stunting prevalence at 7 m, except Fortaleza (2/100, 2%) and Bhaktapur (14/221, 6%).

**Table 4:** Enteropathogen burden scores in diarrheal and non-diarrheal stools, mean total diarrhea episodes and nutritional status at the time of the 7 month blood draw, by site.

Site	N	Enteropathogen scores in diarrheal and non-diarrheal stools and cumulative days with diarrhea [mean (95% CI)]				Nutritional status	
		All pathogens	Bacteria	Parasite	Cumulative days of diarrhea	% Stunted^1^	% Anemic^2^
BGD	200	0.76 (0.70–0.82)	0.64 (0.59–0.7)	0.03 (0.02–0.04)	8.2 (6.9–9.5)	20.0%	47.2%
NEB	221	0.58 (0.54–0.63)	0.49 (0.44–0.53)	0.03 (0.02–0.04)	9.4 (8.0–11)	6.3%	79.2%
INV	216	0.74 (0.69–0.8)	0.63 (0.59–0.68)	0.05 (0.04–0.07)	5.2 (4.2–6.1)	20.4%	51.4%
PKN	258	1.34 (1.27–1.41)	1.01 (0.95–1.06)	0.25 (0.22–0.28)	29.2 (26–33)	NA	71.3%
BRF	100	0.95 (0.83–1.07)	0.88 (0.77–1.00)	0.05 (0.03–0.07)	0.3 (0.1–0.5)	2.0%	45.0%
PEL	213	0.53 (0.48–0.57)	0.36 (0.32–0.40)	0.12 (0.09–0.14)	10.2 (8.6–12)	21.6%	63.4%
TZH	94	1.02 (0.92–1.12)	0.84 (0.75–0.93)	0.10 (0.07–0.13)	3.1 (2.3–4.0)	28.0%	61.7%

NA = Not available for PKN due to biases detected in length measurements during data collection.

1 Stunting defined as LAZ < −2.0.

2 Anemia defined as hemoglobin (adjusted for altitude) <11.0 g/dL.

We evaluated the effect of enteropathogen infection with and without diarrhea on OPV response. Univariate analyses revealed that higher enteropathogen detection scores for all pathogens combined were associated with lower response for both serotypes, while higher parasite detection scores in diarrheal and non-diarrheal stools were associated with lower serotype 3 response (Table 3b, Fig. 3a, Fig. 3b). In addition, the bacterial detection score in diarrheal and non-diarrheal stools at 3 months of age was associated with lower Log_2_ titers and greater risk of failure for both serotypes (not shown). No other enteropathogen scores measured during the neonatal period were associated with serotype 1 or 3 response.

**Fig. 3a:**
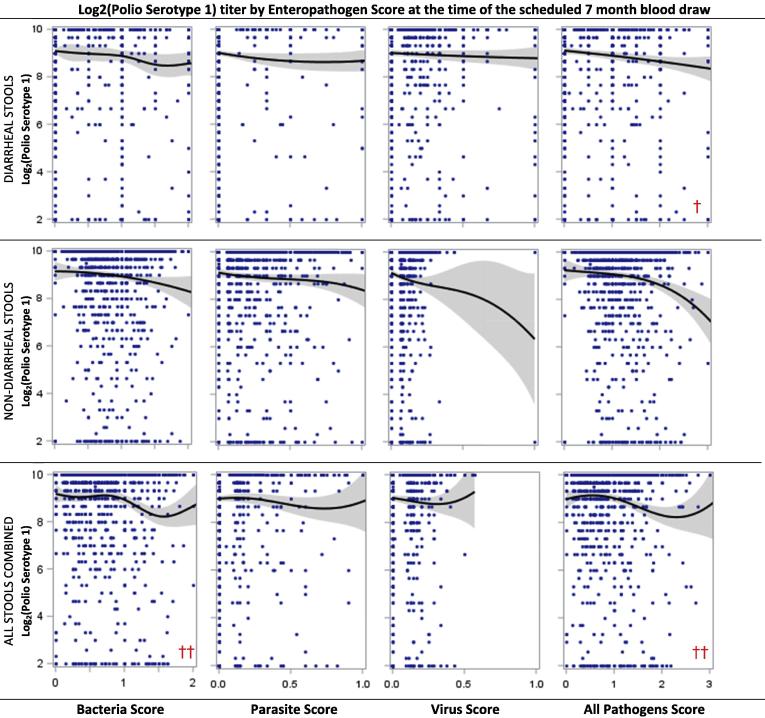
**Polio serotype 1 titers by enteropathogen detection scores (bacteria, parasites, viruses, all combined) computed for diarrheal, non-diarrheal and all stools combined among children receiving at least 3 OPV doses at the scheduled 7 month blood draw.** Scatter plots include penalized B-splines (10 knots) and 95% confidence intervals. A chi-square test was evaluated for each scatter plot comparing the percent of children who failed to seroconvert in a high vs. low enteropathogen score group where high was defined as the approximate 75^th^ percentile of the enteropathogen score (i.e., 1.0, 0.33, 0.15, and 1.25 for Bacteria, Parasite, Virus and All Pathogens Scores, respectively); † indicates p<0.05; †† indicates p<0.01.

**Fig. 3b:**
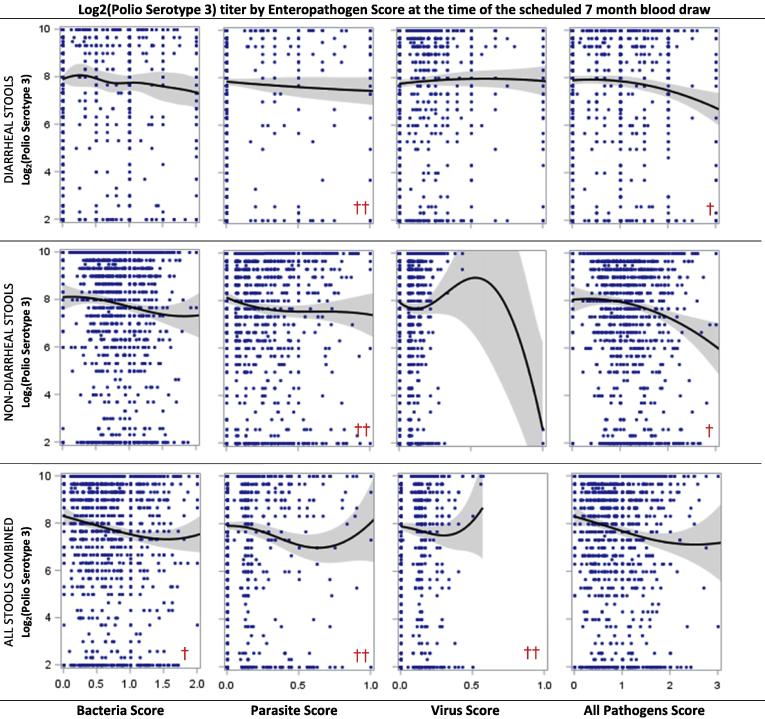
**Polio serotype 3 titers by enteropathogen detection scores (bacteria, parasites, viruses, all combined) computed for diarrheal, non-diarrheal and all stools combined among children receiving at least 3 OPV doses at the scheduled 7 month blood draw.** Scatter plots include penalized B-splines (10 knots) and 95% confidence intervals. A chi-square test was evaluated for each scatter plot comparing the percent of children who failed to seroconvert in a high vs. low enteropathogen score group where high was defined as the approximate 75^th^ percentile of the enteropathogen score (i.e., 1.0, 0.33, 0.15, and 1.25 for Bacteria, Parasite, Virus and All Pathogens Scores, respectively); † indicates p < 0.05; †† indicates p < 0.01.

The final multivariate models indicate that enteropathogen detection in diarrheal, non-diarrheal and both stool types combined were differentially associated with OPV seroconversion failure and titer (Table 2a, Table 2b). Initial model selection (Supplemental Table 3d) identified the importance of non-diarrheal enteropathogen scores primarily associated with serotype 3 response, while diarrheal enteropathogen scores associated with serotype 1. For serotype 1, a one-unit increase in the diarrhea-associated all-pathogen score at the time of blood draw increased the odds failure by 1.9 (95% CI 1.23–2.90; no other enteropathogen scores were associated with serotype 1 failure once this variable was included in the model). In contrast, serotype 1 titer was negatively associated with the combined stool type bacteria score at the blood draw and the diarrhea-associated bacteria score at 3 months of age. For serotype 3, parasite and bacterial scores were predictive of response: a one-unit increase in the three-month bacteria score in combined stool types was associated with a 3.0 (95% CI 1.56–5.78) higher odds of failure and a 0.53 (95% CI 0.29–0.77) lower Log_2_ titer. The non-diarrheal parasite score was associated with a 0.41 (95% CI 0.04–0.77) lower Log_2_ titer, while the combined stool type parasite score increased odds of failure by 7.6 (95% CI 1.51–37.9). As with serotype 1, other enteropathogen scores were related to serotype 3 response, but were not important after adjusting for parasite and bacterial scores.

In contrast to enteropathogen detection scores, diarrhea incidence most individual enteropathogens (*Giardia, EAEC, Cryptosporidium*), and most biomarkers of gut inflammation (α-1 antitryptin, neopterin, myeloperoxidase, lactulose) were not associated with serotype 1 or 3 response (Supplemental Tables 3d and 3e). *Campylobacter* detection in either diarrheal or non-diarrheal stools was associated with increased failure and lower titers for both serotypes; however, it did enter the final models. Adding urinary mannitol z-score at 6 months of age did slightly improve model fit for serotype 3 failure based on AIC; however it also resulted in a loss of 200 observations due to missing data. A one-unit increase in the mannitol z-score was associated with a 0.67 (95% CI 0.41–0.94) lower odds of serotype 3 failure.

Infant feeding practices had a larger influence on serotype 1 response vs. serotype 3. Nearly one fifth (19%) of children were introduced to solid foods before 2 months of age, corresponding with 2.5 higher odds of serotype 1 failure (95% CI 0.83–7.5) and 0.26 lower Log_2_ titer (95% CI 0.01–0.52) compared to introduction after 4 months (Table 2a). Introduction of solids between 2 and 4 months of age increased odds of serotype 1 failure by 4.2 (95% CI 1.6–11.0) and lowered Log2 titer by 0.29 (95% CI 0.07–0.50) compared to introduction after 4 months of age. For serotype 3, no infant feeding practices were associated with response in the final multivariate models or in model selection (Supplemental Table 3b). Duration of breastfeeding (exclusive or partial) was not predictive of response to either serotype.

Measures of nutritional status were not strongly associated with OPV response. Growth velocity, LAZ and WLZ were not associated with response to either serotype (Table 3b, Supplementary Table 3c). Underweight (WAZ < −2) was not associated with serotype 1 response, nor with serotype Log_2_ 3 titer; however, less time considered underweight was related to lower odds of serotype 3 failure (Table 2b). The only nutritional biomarker associated with OPV response was transferrin receptor (TfR). Overall, 58% of children with TfR < 8.3 were anemic compared to 73% of children with TfR > 8.3, which is considered iron-deficiency anemia. Lower TfR concentrations (<2.9 μg/mL) reduced serotype 1 Log_2_ titer by 0.33 (95% CI 0.06–0.60), and reduced serotype 3 Log_2_ titer by 0.41 (95% CI 0.07–0.74) compared to TfR above 8.3 μg/mL (Table 2a, Table 2b).

### 3.3 Socioeconomic status and HOME environment

**Fig. 4:**
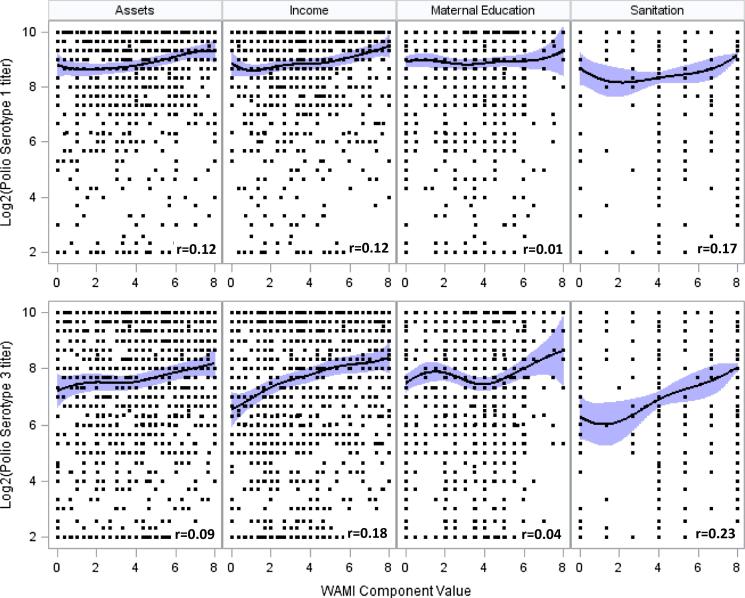
**Serotype 1 and 3 titer distributions and confidence intervals for WAMI component scores at 15 months.** Penalized B-splines were fit using 10 knots. Pearson correlation coefficients (denoted *r*) are reported in the lower right corner of each scatter plot (all correlations have p < 0.0001 for the test of *r* being different from 0, except correlations with Maternal Education, which were not significant)

**Fig. 5:**
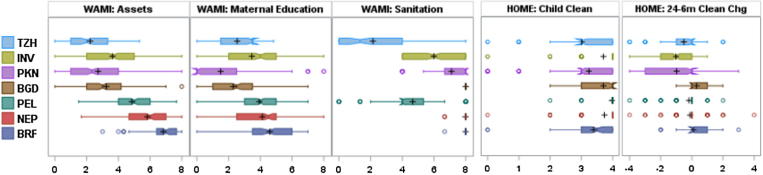
**Distribution of WAMI component and HOME Cleanliness scores by site.** Sites are sorted by highest to lowest overall WAMI score. Symbol descriptions: the box represents the Interquartile Range (25^th^ to 75^th^ percentile); the black plus (+) is the mean; the median is indicated by the box notch; lines extending from the box with hash marks are 1.5 times the interquartile values; and circles represent potential outliers.

WAMI components were strongly predictive of OPV response. Higher serotype 1 titers were associated with higher WAMI component scores in the penalized B-spline models (Fig. 4), but there was no relationship between titers and HOME environment scores (not shown). WAMI and HOME score relationships with serotype 3 were similar to serotype 1. Although WAMI and HOME scores were moderately correlated (Pearson correlation >0.4 for most combinations), differences between WAMI and HOME scores with OPV response are partially explained by their variability across sites (Fig. 4). WAMI components were heterogeneous: Fortaleza and Bhaktapur WAMI scores were significantly higher than in Haydom and Vellore. In contrast, Child Cleanliness scores were homogenous, with some declines observed by 24 months (see Fig. 5).

Multivariate models confirmed strong relationships between WAMI and OPV response, and moderate relationships with HOME scores. Initial model selection identified all WAMI components as significantly related to serotype 3 response, while assets and sanitation were significantly related to serotype 1 (Supplemental Table 3a). AIC differentiated the best fitting models.

For serotype 1, the assets component and overall WAMI index were most predictive of the final failure and Log_2_ titer models, respectively. A higher Assets score was associated with lower odds of failure, but was not significant. A 0.1 unit increase in WAMI was associated with a 0.08 (95% CI 0.077–0.081) Log_2_ titer increase (Table 2a). For serotype 1 titer, Assets and Sanitation were the most predictive components associated with response; however, the WAMI index provided the best model fit. Improved home environment as measured by the Child Cleanliness score (change from 6 to 24 months) and an interaction with age were predictive of serotype 1 failure. In addition, an interaction between age and change in Child Cleanliness Collectively, improving Child Cleanliness was associated with lower odds of serotype 1 failure.

Serotype 3 response was predicted by the WAMI index, but not HOME scores. A 0.1 unit increase in WAMI was associated with 0.72 (95% CI 0.70–0.75) lower odds of failure and 0.123 (95% CI 0.119–0.127) increase in Log_2_ titer (Table 2b). Replacing WAMI with sanitation in the final failure model resulted in similar AIC, with a one-unit change in sanitation resulting in a 0.74 (95% CI 0.60–0.92) lower odds of serotype 3 failure.

## 4 Discussion

The MAL-ED study allowed for the evaluation of factors influencing OPV seroconversion under real-world conditions at a time when strategies and interventions had been implemented to maximize OPV response. Our unique study design differs from most OPV studies particularly in the intensively recorded vaccine administration, child health (enteropathogen and diarrheal surveillance, nutrition, feeding practices), and indicators of home environment and poverty. The design allowed us to test—across multiple sites with varying health systems and socioenvironmental contexts—how OPV immunization programs were performing just prior to the global shift to IPV. In the MAL-ED cohort, six OPV doses maximized response for both serotypes (four minimized serotype 1 serofailure, five minimized serotype 1 serofailure when Naushero Feroze excluded, and five maximized serotype 1 and 3 titers), and OPV response was attenuated by high enteropathogen infection, early introduction of solids, and poor socioeconomic environment.

Both enteropathogen detection and diarrhea occurrence were intensely measured in MAL-ED. Neither diarrhea occurrence, diarrhea at the time of OPV administration, nor individual enteropathogen infections during a diarrhea episode were found to be associated with either polio serotype response. Although diarrhea has been historically identified as a predictor of OPV response (e.g., [28], [29], [51]), the majority of diarrhea experienced by MAL-ED children was of short duration and mild severity. Enteropathogen category scores (bacteria, parasites, all pathogens combined) for each stool type did have differential serotype response relationships with diarrheal-associated scores predictive of serotype 1 and non-diarrheal (and combined stool types) associated with serotype 3. The surprising lack of a dominant signal between diarrhea and OPV response suggests that severe clinical manifestations of infection are not required to exert pernicious influence. This association was particularly found for bacteria and parasites; viral pathogens were not associated with response, possibly due to the limited number of viruses screened.

Increased enteropathogen detection has been correlated with several biomarkers indicating gut inflammation or permeability in this cohort [52], [53]. However, with the exception of mannitol predicting serotype 3 failure, these biomarkers were not associated with OPV response. Biomarkers indicative of gut function show some promise for measuring normal mucosal immune response [54]; however, this is an area of on-going research and other markers may better reflect immune response in children.

During the early years of OPV implementation, transplacental antibody transfer and the intensity of early, exclusive breastfeeding were hypothesized to promote non-responsiveness to OPV [13], [14], [16]. Recent research has indicated that longer breastfeeding duration and improved nutritional status may enhance OPV response [29], [30]. We did not find strong associations between breastfeeding (exclusive or partial) or growth measures and OPV failure. The effect of breastfeeding was likely attenuated given that the median duration of exclusive breastfeeding was under 35 days in Bhaktapur, Loreto, Naushero Feroze, and Haydom, with only children in Dhaka exclusively breastfed for more than 90 days. The heterogeneous feeding practices and growth rates of the MAL-ED cohort potentially improved testing of these factors compared to more homogeneous, single cohort/population studies. Although growth velocity and stunting prevalence were not predictive of OPV response in our cohort, chronic underweight was predictive of serotype 3 response, consistent with previous research [29]. TfR was predictive of serotype 1 and 3 titer; however, further analyses did not identify anemia or iron-deficiency anemia as predictors.

Our findings strongly support ancillary benefits of poverty reduction. Our measure of socioeconomic status (WAMI) was consistently a strong predictor of OPV response. In sites that shared similar vaccine schedules, OPV response tended to be significantly higher in those with better WAMI scores. Conceptually, socioeconomic status is predictive of quality of the HOME environment, enteropathogen detection and incidence of diarrhea [55], [56]; yet only enteropathogen detection was predictive of OPV response. However, it is enlightening that improved child cleanliness was associated with lower serotype 1 failure, which was indicative of both overall home cleanliness and positive home hygienic behaviour.

Our results highlight a key challenge for the WHO Polio Endgame Strategy of achieving >80% vaccine coverage with full immunogenicity. Seroconversion rates in the MAL-ED cohort were similar to previous reports from low- and middle-income countries [19], [57], [58], with approximately 10% non-response following three OPV doses. However, at 7 months of age, 14% of children did not receive three OPV doses, making the 80% threshold goal tenuous. Even in SAV, where both OPV and IPV are administered, only 70% of children were fully vaccinated by 7 months (MAL-ED OPV-IPV companion paper). A key factor in improved coverage is socioeconomic status. It is imperative to recognize that the increased cost of IPV may result in unintended lower coverage. Although supplemental dosing of IPV with OPV may overcome challenges regarding coverage and improved immunogenicity [33], [59], our findings suggest that responses to OPV-IPV schedules may improve as more children rise out of poverty. Improved protection against polio could be factored into cost-effectiveness evaluations of interventions aimed at eradicating poverty. Conditional cash-transfer programs provide some evidence of this, with many of these poverty reduction programs impacting household consumption, labor decisions and child health [60], [61], [62]. Whether OPV, IPV or other vaccine responses improve as an externality to poverty reduction interventions is an important area for further research.

Study limitations include the inability to evaluate serotype 2 interference (likely minimal due to current trivalent OPV formulation) [19], [20], the inability to assess mucosal immune responses preventing transmission, and the lack of data on maternal antibodies. Although the neutralizing antibodies measured in this study reflect individual protection against poliomyelitis, the prevalence of lack of seroconversion combined with inadequate coverage suggest there is substantial risk for transmission [63,64]. Most importantly, the observational nature of the study led to limitations (and strengths) arising from variation in OPV dosing and timing of blood collection. Since MAL-ED was designed to test multiple hypotheses that required repeated collection of data from children on a bi-weekly basis, blood collection for vaccine response was limited to two time points.

MAL-ED is one of the most comprehensive multi-site enteric disease birth cohort studies conducted. Sites exhibited heterogeneity in several respects, most notably that OPV non-response was greater at the lower SES sites. However, no site-level or age-period differences were detected in the associations between the factors of greatest importance for OPV failure. This consistency provides clear guidance for improving response to serotypes 1 and 3, which can inform implementation of the WHO’s Global Polio Eradication Initiative Plan. Our results suggest that the most important pathways to achieve OPV seroprotection in impoverished settings include administering the minimally optimal number of vaccine doses and improving socio-environmental conditions and sanitation to reduce early enteropathogen exposure.

## 5 Contributors

William K. Pan devised the model in discussion with Carl J. Mason, Jessica C. Seidman, Christel Hoest, Stacey L. Knobler, Dinesh Mondal, Asad Ali, and Pascal Bessong. William K. Pan ran the analysis. William K. Pan, Carl J. Mason, Jessica C. Seidman, Asad Ali, Dinesh Mondal and Christel Hoest participated in the interpretation of the results and drafted the manuscript. The MAL-ED Investigators participated in the design, conduct, and analysis of the MAL-ED study and its results.

## Conflict of interest

We declare that we have no conflicts of interest. The findings and conclusions in this report are those of the authors and do not necessarily represent the official position of the US National Institutes of Health or Department of Health and Human Services.

## Funding

The Bill & Melinda Gates Foundation.
