# Supplementary material for: Oral polio vaccine response in the MAL-ED birth cohort study: Considerations for polio eradication strategies
Source: Vaccine. 2019 Jan 7;37(2):352–65. doi: 10.1016/j.vaccine.2018.05.080 (PMC6325791; doi:10.1016/j.vaccine.2018.05.080)
Supplement: Supplementary data 1 [file mmc1.docx]

| **Category** | **Concept to capture** | **Variables defined** |
| --- | --- | --- |
| VACCINATION HISTORY | Total OPV doses | Doses received by the blood draw (range 3-12)  Doses received by week 4, 8, 12, etc.^iv^ |
|  | Schedule^i^ | Age at first (second, third, etc.) dose  Intervals (days) between doses 1 and 2, 2 and 3, etc.  Days since last dose  Doses received by week 4, 8, 12, etc. |
|  | Seasonality | Doses received during the rainy season |
| FEEDING PATTERNS | Breastmilk intake | Duration of exclusive breastfeeding (defined as breastmilk as the main and only source of food with inclusion of medicines, vitamins and minerals)  Duration of partial or predominant breastfeeding (Predominant is defined as breastmilk is a predominant source of milk to the child with inclusion of water, water-based liquids like teas and juices, vitamins and medicine; Partial is defined as a child consuming breastmilk in addition to consumption of any food or liquids)  Breastfeeding status to week 4, 8, 12, etc.  Breastfeeding status during OPV dose 1, 2, 3 |
|  | Intake of solids or other liquids | Age when solids introduced  Age when milk introduced  Days before OPV dose that solids (milk) introduced  Solids (milk) introduced by week 4, 8, 12, etc. |
| GROWTH & NUTRIENT STATUS | Anthropometry ^ii^ | LAZ, WAZ, WLZ – changes over time, status by month  Days and % of months stunted/wasted/underweight to: OPV dose; Month #; Blood draw  Stunted/Wasted/Underweight during OPV dose |
|  | Plasma biomarkers of nutrient status (7, 15 months) | Zinc  Retinol  Transferrin receptor (TFR) |
| ENTERIC INFECTION | Diarrhoea symptoms | Cumulative diarrhoea episodes to: blood draw, OPV dose, week 4, 8, 12, etc.  Total days of diarrhoea to: blood draw, OPV dose, week 4, 8, 12, etc.  Diarrhoea 1, 3, or 5 days before (or after) OPV dose 1, 2, 3 |
|  | Pathogen detection^iii^ | Pathogen / Bacteria / Virus / Parasite score^iii^  Score (above) to week 4, 8, 12, etc.  Score (above) exclude Campylobacter, Cryptosporidium, Giardia and EAEC  Score of Campylobacter (or Cryptosporidium, Giardia or EAEC) to blood draw or by month  Diarrhoea-associated pathogen scores |
|  | Fecal and Urinary biomarkers of gut function | Myeloperoxidase (MPO): fecal biomarker, included in the model as Log value of the mean over months 1-3 and over months 1-7  Neopterin (NEO): fecal biomarker, same definition as MPO  α-1-Antitrypsin (AAT): fecal biomarker, included in the model as (1) categorical using 0.5mg/g as a cutoff and (2) mean measure for months 1-3 and 1-7  Lactulose: urinary biomarker (measured at 3, 6, 9 and 15 months), included in the model using z-scores estimated at 3m, 6m and mean for 3 and 6 months  Mannitol: urinary biomarker, same definition as Lactulose  Lactulose:Mannitol Ratio: Same definition as Lactulose |
| HOME ENVIRONMENT | WAMI Index-SES measures | ASSETS – Asset score composed of mattress, chair, table, television, and refrigerator ownership, bank account holder, kitchen in household, and fewer than 2 people per room density  SCHOOLING – Educational score, computed as the years of maternal education divided by two  INCOME – Reported income in US dollars, categorized in noniles  SANITATION – Sum of sanitation (toilet type) and drinking water source  WAMI – Composite score for Water (sanitation), Assets, Maternal education, & Income |
|  | HOME Factors | Clean and Safe Environment – factor comprised of 5 items: Stove in safe area; Play area free of hazards; House relatively light; House neat and orderly; Taken to health clinic regularly  Child Cleanliness – factor comprised of 4 items: Child clean; Clothes clean; Hair neat / washed recently; House neat and orderly |

**Supplemental Table 1. Parameterization of variables evaluated against OPV response by category.**

i. Interval refers to days between doses.

ii. Anthropometry measures over time were quantified by using the monthly surveillance data and carrying the value forward until the next measure. Analyses involving LAZ, WLZ and growth velocity excluded PKN due to inconsistencies identified in field data collection at that site.

iii. Scores of pathogen, bacteria, virus, and parasite detection are cumulative counts of detection divided by the number of stools collected to that point in time.

iv. Weeks 4, 8, 12, etc., correspond to roughly months 1, 2, 3, etc.; however, times measured in months refer to data collected on the day of the month of the child’s birthday (eg, a child born October 20, will have data collected on November 20, December 20, etc.).

|  |  | **Serotype 1 Failure** | | | **Serotype 3 Failure** | | | | **Log_2_ Serotype 1 titer ^2^** | | | **Log_2_ Serotype 3 titer ^2^** | | |
| --- | --- | --- | --- | --- | --- | --- | --- | --- | --- | --- | --- | --- | --- | --- |
|  |  | **Odds Ratio** |  | **(95% CI)** | **Odds Ratio** |  | **(95% CI)** | | **Beta** |  | **(95% CI)** | **Beta** |  | **(95% CI)** |
| **Intercept** | (log odds) | -5.9589 |  |  | -5.9117 |  |  |  | 9.1 | ^†††^ | (8.73-9.46) | 7.8 | ^†††^ | (7.34-8.31) |
| **Site** (REF = BRF) | BGD | 2.15 |  | (0.27-16.9) | 4.51 |  | (0.64-31.9) | | -0.37 | ^*^ | (-0.78-0.04) | -0.24 |  | (-0.79-0.31) |
|  | INV | 6.04 |  | (0.66-54.99) | 11.6 | ^†^ | (1.46-91.7) | | -1.01 | ^†††^ | (-1.44--0.57) | -1.62 | ^†††^ | (-2.2--1.03) |
|  | NEB | 1.07 |  | (0.13-8.9) | 4.47 |  | (0.67-29.9) | | -0.2 |  | (-0.56-0.17) | -0.19 |  | (-0.68-0.31) |
|  | PEL | 4.96 |  | (0.69-35.73) | 10 | ^†^ | (1.47-68.04) | | -0.42 | ^†^ | (-0.83--0.01) | -0.03 |  | (-0.58-0.51) |
|  | PKN | 9.44 | ^*^ | (0.9-99.43) | 21.4 | ^††^ | (2.53-180.9) | | -0.48 | ^†^ | (-0.91--0.04) | -0.53 | ^*^ | (-1.12-0.05) |
|  | TZH | 1.99 |  | (0.16-25.57) | 8.77 | ^*^ | (0.81-95.4) | | -0.01 |  | (-0.6-0.57) | -0.76 | ^*^ | (-1.54-0.02) |
| **Age at blood draw** (in months) ^1^ |  | 0.99 |  | (0.92-1.07) | 1.01 |  | (0.95-1.07) | | -0.03 | ^†††^ | (-0.05--0.01) | -0.04 | ^†††^ | (-0.06--0.03) |
| **OPV** **doses** (REF = 3) | 4 | 0.26 | ^††^ | (0.09-0.73) | 0.35 | ^††^ | (0.16-0.77) | | 0.31 | ^†††^ | (0.09-0.52) | 0.42 | ^††^ | (0.16-0.68) |
|  | 5+ | 0.09 | ^†††^ | (0.02-0.32) | 0.10 | ^†††^ | (0.04-0.26) | | 0.58 | ^††^ | (0.34-0.82) | 0.90 | ^†††^ | (0.6-1.2) |
| **WAMI** (overall index) ^1^ |  | 0.06 | ^†^ | (0-0.99) | 0.01 | ^†††^ | (0-0.15) | | 0.91 | ^††^ | (0.26-1.55) | 1.71 | ^†††^ | (0.84-2.59) |
| **Supplemental Table 2. Base Models for Serotype 1 and 3 Seroconversion Failure and Log_2_ titers**. These two sets of multivariate regression models fit seroconversion failure (columns 1 and 2) and Log_2_ serotype titers (columns 3 and 4), adjusted for child within site random effects. This set of covariates (site, age, OPV doses, WAMI) is included in each model to evaluate effect sizes reported in Supplemental Tables 3a-3e, which are used to identify the final models reported in Tables 2 and 3. Supplemental Tables 2a-2e represent covariates defined to capture the effect of a particular theme (e.g., socioeconomic status and home environment, infant feeding behavior, nutritional status, enteric infection and gut function). The AIC for each model is, respectively (columns 1-4): 806.52, 1521.4, 9495.2, and 10683. The sample size (complete non-missing data) for each of these base models is 2541. | | | | | | | | | | | | | | |
| ††† p<0.001; †† p<0.01; † p<0.05; * p<0.10; | | | | | | | | | | | | | | |
| 1 - Age was centered at 7 months, WAMI is centered at 0.5 (range 0-1) | | | | | | | | | | | | | | |

|  |  | **Serotype 1 Failure** | | | **Serotype 3 Failure** | | | **Log_2_ Serotype 1 titer** | | | **Log_2_ Serotype 3 titer** | | |
| --- | --- | --- | --- | --- | --- | --- | --- | --- | --- | --- | --- | --- | --- |
|  |  | **Odds Ratio** | **(SE)^3^** |  | **Odds Ratio** | **(SE)^3^** |  | **Beta** | **(SE)** |  | **Beta** | **(SE)** |  |
| **WAMI Components^1^** |  |  |  |  |  |  |  |  |  |  |  |  |  |
| Water and Sanitation |  | 0.89 | (0.12) |  | 0.73 | (0.11) | ^††^ | 0.096 | (0.033) | ^††^ | 0.13 | (0.044) | ^††^ |
| Household Assets |  | 0.76 | (0.12) | ^†^ | 0.78 | (0.1) | ^†^ | 0.09 | (0.027) | ^†††^ | 0.10 | (0.036) | ^††^ |
| Maternal Education |  | 0.94 | (0.11) |  | 0.82 | (0.1) | ^†^ | 0.024 | (0.026) |  | 0.10 | (0.035) | ^††^ |
| Household Income |  | 0.84 | (0.12) |  | 0.8 | (0.11) | ^†^ | 0.013 | (0.028) |  | 0.08 | (0.038) | ^†^ |
| **HOME Environment^2^** |  |  |  |  |  |  |  |  |  |  |  |  |  |
| Safe Environment |  | 0.85 | (0.22) |  | 0.85 | (0.19) |  | 0.079 | (0.053) |  | 0.04 | (0.072) |  |
| Child Cleanliness |  | 1.02 | (0.27) |  | 0.74 | (0.23) |  | 0.032 | (0.066) |  | 0.166 | (0.089) | ^*^ |
| Change in cleanliness (24m vs 6m) | | 0.7 | (0.16) | ^†^ | 1.12 | (0.15) |  | 0.13 | (0.044) | ^††^ | 0.047 | (0.059) |  |
| **Supplemental Table 3a. Base Model covariates plus Socioeconomic Status (WAMI) components and Home Environment measures.** WAMI components replaced the WAMI covariate in the Base model, HOME measures were added to the Base model. Each estimate from the table is from a separate model. WAMI Assets provided a better model fit for Serotype 1 failure than WAMI, thus WAMI Assets are used in subsequent Base models. | | | | | | | | | | | | | |
| ††† p<0.001; †† p<0.01; † p<0.05; * p<0.10;  1 - All WAMI components range from 0-8 and are centered at 4  2 - HOME Safe Environment and Child Cleanliness range from 0-4; Change in cleanliness ranges from -4 to 4  3 - Standard error of the log odds, not the OR | | | | | | | | | | | | | |

|  |  | **Serotype 1 Failure** | | | **Serotype 3 Failure** | | | **Log_2_ Serotype 1 titer** | | | **Log_2_ Serotype 3 titer** | | |
| --- | --- | --- | --- | --- | --- | --- | --- | --- | --- | --- | --- | --- | --- |
|  |  | **Odds Ratio** | **(SE)^1^** |  | **Odds Ratio** | **(SE)^1^** |  | **Beta** | **(SE)** |  | **Beta** | **(SE)** |  |
| **Months Exclusive Breastfeeding** (REF >4m) | 2-4m | 1.09 | (0.67) |  | 0.83 | (0.59) |  | -0.003 | (0.154) |  | -0.02 | (0.208) |  |
|  | <2m | 1.77 | (0.63) |  | 0.84 | (0.56) |  | -0.155 | (0.145) |  | -0.012 | (0.196) |  |
| **Age milk introduced** (REF >4m) | 2-4m | 1.3 | (0.55) |  | 1.84 | (0.49) |  | -0.169 | (0.129) |  | -0.308 | (0.175) | ^*^ |
|  | <2m | 1.76 | (0.45) |  | 1.71 | (0.42) |  | -0.224 | (0.113) | ^†^ | -0.284 | (0.152) | ^*^ |
| **Age milk or solids introduced with breastmilk** (REF >4m) | 2-4m | 1.98 | (0.52) |  | 1.46 | (0.47) |  | -0.305 | (0.124) | ^†^ | -0.197 | (0.168) |  |
|  | <2m | 3.28 | (0.48) | ^†^ | 1.44 | (0.44) |  | -0.403 | (0.117) | ^†††^ | -0.287 | (0.158) | ^*^ |
| **Age solids introduced** (REF >4m) | 2-4m | 3.61 | (0.47) | ^††^ | 1.11 | (0.41) |  | -0.346 | (0.111) | ^††^ | -0.103 | (0.15) |  |
|  | <2m | 3.08 | (0.54) | ^†^ | 1.08 | (0.5) |  | -0.345 | (0.133) | ^††^ | -0.221 | (0.18) |  |
| **Age weaned** (REF >24m) | 12-24m | 1.37 | (0.48) |  | 1.19 | (0.43) |  | -0.105 | (0.112) |  | 0.035 | (0.15) |  |
|  | <12m | 1.65 | (0.7) |  | 5.34 | (0.65) | ^††^ | -0.259 | (0.175) |  | -0.624 | (0.235) | ^††^ |
| **Supplemental Table 3b. Base Model covariates plus Infant Feeding Behavior.** Infant feeding behavior was recorded during biweekly interviews and monthly surveillance. Anthropometry is measured monthly. | | | | | | | | | | | | | |
| ††† p<0.001; †† p<0.01; † p<0.05; * p<0.10;  1 - Standard error of the log odds, not the OR | | | | | | | | | | | | | |

|  |  | **Serotype 1 Failure** | | | **Serotype 3 Failure** | | | **Log_2_ Serotype 1 titer** | | | **Log_2_ Serotype 3 titer** | | |
| --- | --- | --- | --- | --- | --- | --- | --- | --- | --- | --- | --- | --- | --- |
|  |  | **Odds Ratio** | **(SE)^1^** |  | **Odds Ratio** | **(SE)^1^** |  | **Beta** | **(SE)** |  | **Beta** | **(SE)** |  |
| **Retinol** <20 |  | 1.01 | (0.39) |  | 0.8 | (0.32) |  | -0.047 | (0.080) |  | 0.066 | (0.100) |  |
| **Retinol** |  | 1.01 | (0.02) |  | 1.01 | (0.017) |  | 0.0004 | (0.004) |  | -0.001 | (0.005) |  |
| **Ferritin** <12 |  | 1.82 | (0.37) |  | 1.28 | (0.31) |  | -0.089 | (0.083) |  | 0.054 | (0.104) |  |
| **Ferritin** |  | 0.99 | (0.01) |  | 1 | (0.003) |  | 0.001 | (0.001) |  | -0.001 | (0.001) |  |
| **Transferrin Receptor** (REF TFR<2.9) | 2.9≤TFR≤8.3 | 0.53 | (0.5) |  | 0.91 | (0.42) |  | 0.187 | (0.122) |  | 0.25 | (0.148) | ^*^ |
|  | TFR>8.3 | 0.57 | (0.6) |  | 0.7 | (0.49) |  | 0.363 | (0.138) | ^††^ | 0.436 | (0.169) | ^††^ |
| **Transferrin Receptor** |  | 0.98 | (0.05) |  | 0.96 | (0.03) |  | 0.016 | (0.009) | ^*^ | 0.015 | (0.011) |  |
| **Zinc <9.9mmL** |  | 1.11 | (0.41) |  | 1.12 | (0.33) |  | 0.008 | (0.008) |  | 0.049 | (0.108) |  |
| **Zinc** |  | 0.99 | (0.04) |  | 0.99 | (0.03) |  | 0.0005 | (0.009) |  | 0.0017 | (0.011) |  |
| **Alpha-1-Acid Glycoprotein >100** |  | 1.01 | (0.33) |  | 0.86 | (0.26) |  | 0.036 | (0.072) |  | 0.118 | (0.088) |  |
| **Alpha-1-Acid Glycoprotein** |  | 0.996 | (0.005) |  | 0.997 | (0.003) |  | -0.0002 | (0.001) |  | 0.002 | (0.001) |  |
| **Hemoglobin (g/dL)** |  | 0.86 | (0.12) |  | 1.12 | (0.10) |  | 0.018 | (0.027) |  | -0.032 | (0.033) |  |
| **LAZ at collection** |  | 1.01 | (0.18) |  | 0.798 | (0.17) |  | 0.044 | (0.044) |  | 0.081 | (0.057) |  |
| **WHZ at collection** |  | 1.2 | (0.16) |  | 0.94 | (0.14) |  | 0.004 | (0.038) |  | 0.055 | (0.049) |  |
| **WAZ at collection** |  | 1.19 | (0.17) |  | 0.87 | (0.15) |  | 0.023 | (0.042) |  | 0.088 | (0.054) |  |
| **% days underweight** (birth to blood draw, REF ≥50%) | 10-49% | 1.22 | (0.63) |  | 0.29 | (0.52) | ^†^ | 0.065 | (0.153) |  | 0.3 | (0.193) |  |
|  | <10% | 0.79 | (0.57) |  | 0.22 | (0.48) | ^††^ | 0.211 | (0.14) |  | 0.436 | (0.182) | ^†^ |
| **% days stunted** (birth to blood draw, REF ≥50%) | 10-49% | 0.71 | (0.47) |  | 0.35 | (0.46) | ^†^ | 0.131 | (0.131) |  | 0.498 | (0.163) | ^††^ |
|  | <10% | 0.45 | (0.45) | * | 0.67 | (0.42) |  | 0.156 | (0.123) |  | 0.19 | (0.16) |  |
| **% days wasted** (birth to blood draw, REF ≥50%) | 10-49% | 0.88 | (0.92) |  | 0.74 | (0.67) |  | 0.236 | (0.212) |  | 0.344 | (0.268) |  |
|  | <10% | 1.1 | (0.87) |  | 0.29 | (0.67) | ^*^ | 0.385 | (0.206) | ^*^ | 0.587 | (0.265) |  |
| **Supplemental Table 3c. Base Model covariates plus indicators of nutritional status.** Serum biomarkers (retinol, TFR, zinc, A1A glycoprotein, hemoglobin) are measured concurrently with vaccine response, anthropometry is monthly. Percent of days underweight, stunted or wasted assumes last value carried forward, therefore if a child is considered stunted during one particular month, they remain stunted until the next measure indicates they are no longer stunted. | | | | | | | | | | | | | |
| ††† p<0.001; †† p<0.01; † p<0.05; * p<0.10;  1 - Standard error of the log odds, not the OR | | | | | | | | | | | | | |

|  |  | **Serotype 1 Failure** | | | **Serotype 3 Failure** | | | **Log_2_ Serotype 1 titer** | | | **Log_2_ Serotype 3 titer** | | |
| --- | --- | --- | --- | --- | --- | --- | --- | --- | --- | --- | --- | --- | --- |
|  |  | **Odds Ratio** | **(SE)^1^** |  | **Odds Ratio** | **(SE)^1^** |  | **Beta** | **(SE)** |  | **Beta** | **(SE)** |  |
| **Diarrhea episodes to age:** | 3m | 1.03 | (0.02) |  | 0.98 | (0.02) |  | -0.004 | (0.007) |  | 0.008 | (0.009) |  |
|  | 4m | 1.03 | (0.02) |  | 0.99 | (0.02) |  | -0.006 | (0.005) |  | 0.002 | (0.007) |  |
|  | 5m | 1.02 | (0.01) | ^*^ | 0.99 | (0.01) |  | -0.005 | (0.004) |  | 0 | (0.005) |  |
| **Cumulative days of diarrhea:** | to blood draw | 1.003 | (0.01) |  | 0.99 | (0.01) |  | 0.001 | (0.002) |  | 0.001 | (0.003) |  |
|  | before OPV d2 | 1 | (0.03) |  | 0.99 | (0.03) |  | 0 | (0.007) |  | 0.002 | (0.01) |  |
|  | before OPV d3 | 1.01 | (0.02) |  | 0.99 | (0.02) |  | -0.003 | (0.005) |  | 0.001 | (0.007) |  |
| **Diarrhea occurrence within 3 days of:** | OPV1 | 2.37 | (0.62) |  | 1.53 | (0.61) |  | -0.202 | (0.175) |  | -0.143 | (0.237) |  |
|  | OPV2 | 1.11 | (0.47) |  | 0.63 | (0.46) |  | 0.047 | (0.125) |  | 0.159 | (0.169) |  |
|  | OPV3 | 1.59 | (0.41) |  | 0.74 | (0.4) |  | -0.192 | (0.109) | ^*^ | -0.092 | (0.148) |  |
| **Enteropathogen scores, diarrhea & non-diarrheal stools** | | | | | | | | |  |  |  |  |  |
| All pathogens combined | at blood draw | 3.05 | (0.42) | ^††^ | 2.39 | (0.36) | ^†^ | -0.432 | (0.099) | ^†††^ | -0.347 | (0.128) | ^††^ |
|  | at 3m of age | 1.19 | (0.31) |  | 2.83 | (0.29) | ^†††^ | -0.153 | (0.08) | ^*^ | -0.457 | (0.108) | ^†††^ |
| Bacteria score | at blood draw | 2.995 | (0.5) | ^†^ | 1.83 | (0.44) |  | -0.507 | (0.119) | ^†††^ | -0.341 | (0.154) | ^†^ |
|  | at 3m of age | 0.96 | (0.38) |  | 3.21 | (0.34) | ^†††^ | -0.155 | (0.091) | ^*^ | -0.534 | (0.122) | ^†††^ |
| Parasite score | at blood draw | 4.43 | (0.94) |  | 8.77 | (0.82) | ^††^ | -0.329 | (0.242) |  | -0.526 | (0.309) | ^*^ |
|  | at 3m of age | 1.99 | (0.73) |  | 1.96 | (0.71) |  | -0.054 | (0.213) |  | -0.224 | (0.287) |  |
| Virus Score | at blood draw | 7.843 | (1.81) |  | 3.406 | (1.58) |  | -0.532 | (0.456) |  | -0.468 | (0.582) |  |
|  | at 3m of age | 2.23 | (1.05) |  | 2.29 | (0.98) |  | -0.267 | (0.293) |  | -0.151 | (0.392) |  |
| Campylobacter | at blood draw | 1.17 | (0.72) | ^†^ | 2.06 | (0.52) |  | -0.354 | (0.157) | ^†^ | -0.457 | (0.193) | ^†^ |
| Cryptosporidium | at blood draw | 0.55 | (1.08) | ^†^ | 0.22 | (0.89) | ^*^ | 0.409 | (0.212) | ^†^ | 0.5 | (0.262) | ^*^ |
| EAEC | at blood draw | 0.638 | (0.64) |  | 1.025 | (0.46) |  | -0.2 | (0.131) |  | -0.296 | (0.16) | ^*^ |
| Giardia | at blood draw | 1.2 | (0.93) | ^*^ | 3.82 | (0.76) | ^*^ | -0.426 | (0.235) | ^*^ | -0.844 | (0.287) | ^††^ |
| **Enteropathogen scores, diarrhea stools** | | | |  |  |  |  |  |  |  |  |  |  |
| All pathogens combined | at blood draw | 1.77 | (0.21) | ^††^ | 1.15 | (0.18) |  | -0.136 | (0.052) | ^††^ | -0.054 | (0.066) |  |
|  | at 3m of age | 1.798 | (0.3) | ^†^ | 1.825 | (0.28) | ^†^ | -0.281 | (0.084) | ^†††^ | -0.295 | (0.115) | ^††^ |
| Bacteria score | at blood draw | 1.74 | (0.26) | ^†^ | 1.09 | (0.24) |  | -0.169 | (0.067) | ^†^ | -0.078 | (0.085) |  |
|  | at 3m of age | 2.28 | (0.36) | ^†^ | 2.14 | (0.35) | ^†^ | -0.386 | (0.102) | ^†††^ | -0.402 | (0.14) | ^††^ |
| Parasite score | at blood draw | 2.622 | (0.61) |  | 2.29 | (0.54) |  | -0.05 | (0.159) |  | -0.007 | (0.2) |  |
|  | at 3m of age | 1.33 | (1.07) |  | 1.785 | (1.03) |  | -0.006 | (0.301) |  | 0.017 | (0.41) |  |
| Virus Score | at blood draw | 1.57 | (0.76) |  | 0.52 | (0.66) |  | -0.051 | (0.188) |  | 0.215 | (0.228) |  |
|  | at 3m of age | 1.23 | (1.12) |  | 2.412 | (0.99) |  | -0.317 | (0.324) |  | -0.419 | (0.439) |  |
| Campylobacter | at blood draw | 1.27 | (0.8) |  | 1.05 | (0.66) |  | 0.032 | (0.182) |  | -0.058 | (0.222) |  |
| Cryptosporidium | at blood draw | 0.27 | (2.48) |  | 0.15 | (1.57) |  | 0.716 | (0.439) | ^*^ | 0.421 | (0.534) |  |
| EAEC | at blood draw | 1.89 | (0.96) |  | 1.53 | (0.76) |  | 0.008 | (0.221) |  | -0.476 | (0.27) | ^*^ |
| Giardia | at blood draw | 0.362 | (1.03) |  | 2.6 | (0.8) |  | 0.162 | (0.23) |  | 0.008 | (0.281) |  |
| **Enteropathogen scores, non-diarrhea stools** | | | |  |  |  |  |  |  |  |  |  |  |
| All pathogens combined | at blood draw | 1.57 | (0.26) | ^*^ | 1.6 | (0.2) | ^†^ | -0.163 | (0.059) | ^††^ | -0.202 | (0.073) | ^††^ |
|  | at 3m of age | 1.08 | (0.29) |  | 2.54 | (0.27) | ^†††^ | -0.118 | (0.074) |  | -0.407 | (0.099) | ^†††^ |
| Bacteria score | at blood draw | 1.297 | (0.35) |  | 1.36 | (0.26) |  | -0.125 | (0.073) | ^*^ | -0.176 | (0.09) | ^†^ |
|  | at 3m of age | 0.76 | (0.37) |  | 2.62 | (0.32) | ^††^ | -0.084 | (0.086) |  | -0.445 | (0.116) | ^†††^ |
| Parasite score | at blood draw | 2.45 | (0.56) |  | 2.29 | (0.49) | ^*^ | -0.277 | (0.147) | ^*^ | -0.403 | (0.18) | ^†^ |
|  | at 3m of age | 1.96 | (0.7) |  | 1.838 | (0.68) |  | -0.044 | (0.203) |  | -0.23 | (0.274) |  |
| Virus Score | at blood draw | 1.28 | (1.11) |  | 2.01 | (0.82) |  | -0.138 | (0.245) |  | -0.275 | (0.304) |  |
|  | at 3m of age | 2.46 | (1.07) |  | 3.026 | (0.98) |  | -0.225 | (0.297) |  | -0.181 | (0.398) |  |
| Campylobacter | at blood draw | 1.08 | (0.71) |  | 3 | (0.6) | ^*^ | -0.172 | (0.17) |  | -0.302 | (0.229) |  |
| Cryptosporidium | at blood draw | 1.614 | (0.74) |  | 1.39 | (0.72) |  | 0.178 | (0.197) |  | 0.055 | (0.266) |  |
| EAEC | at blood draw | 0.539 | (0.53) |  | 2.89 | (0.44) | ^†^ | 0.026 | (0.121) |  | -0.391 | (0.163) | ^†^ |
| Giardia | at blood draw | 0.82 | (1.61) |  | 8.61 | (1.33) |  | -0.157 | (0.415) |  | -1.006 | (0.56) | ^*^ |
| All pathogens combined | at blood draw | 1.57 | (0.26) | ^*^ | 1.6 | (0.2) | ^†^ | -0.163 | (0.059) | ^††^ | -0.202 | (0.073) | ^††^ |
| **Supplemental Table 3d. Base Model covariates plus Diarrhea Prevalence and Enteropathogen Detection Scores in Diarrheal and Non-Diarrheal Stools.** Each enteropathogen score is computed as the sum of specific pathogens detected in stools (by type) up to a specific age divided by the number of stools collected of that stool type to the same age. Enteropathogen scores were computed at the time of the blood draw and up to 1, 2, 3 and 4 months of age. Only the 3^rd^ month of age is reported here. | | | | | | | | | | | | | |
| ††† p<0.001; †† p<0.01; † p<0.05; * p<0.10;  1 - Standard error of the log odds, not the Odds Ratio | | | | | | | | | | | | | |

|  |  | **Serotype 1 Failure** | | | **Serotype 3 Failure** | | | **Log_2_ Serotype 1 titer** | | | **Log_2_ Serotype 3 titer** | | |  |
| --- | --- | --- | --- | --- | --- | --- | --- | --- | --- | --- | --- | --- | --- | --- |
|  |  | **Odds Ratio** | **(SE)^1^** |  | **Odds Ratio** | **(SE)^1^** |  | **Beta** | **(SE)** |  | **Beta** | **(SE)** |  |  |
| **Alpha-1 Antitrypsin** |  |  |  |  |  |  |  |  |  |  |  |  |  |  |
| 1-3 month mean, ALA<0.5mg/g |  | 1.218 | (0.38) |  | 1.886 | (0.35) | ^*^ | -0.077 | (0.095) |  | -0.164 | (0.126) |  |  |
| 1-3 month mean (mg/g) |  | 0.51 | (0.45) |  | 0.516 | (0.37) | ^*^ | -0.164 | (0.094) | ^*^ | -0.076 | (0.127) |  |  |
| 1-7 month mean, ALA<0.5mg/g |  | 1.645 | (0.38) |  | 1.256 | (0.35) |  | 0.052 | (0.093) |  | 0.207 | (0.124) | ^*^ |  |
| 1-7 month mean (mg/g) |  | 0.43 | (0.58) |  | 0.711 | (0.48) |  | 0.124 | (0.125) |  | 0.127 | (0.17) |  |  |
| **Log(Neopterin)** |  |  |  |  |  |  |  |  |  |  |  |  |  |  |
| 1-3 month mean |  | 0.678 | (0.25) |  | 0.717 | (0.22) |  | 0.066 | (0.061) |  | 0.074 | (0.081) |  |  |
| 1-7 month mean |  | 0.509 | (0.35) | ^*^ | 0.683 | (0.31) |  | 0.073 | (0.082) |  | -0.014 | (0.111) |  |  |
| **Log(Myeloperoxidase)** |  |  |  |  |  |  |  |  |  |  |  |  |  |  |
| 1-3 month mean |  | 0.845 | (0.21) |  | 1.14 | (0.19) |  | 0.015 | (0.05) |  | -0.023 | (0.067) |  |  |
| 1-7 month mean |  | 0.92 | (0.31) |  | 1.23 | (0.28) |  | 0.021 | (0.072) |  | -0.028 | (0.097) |  |  |
| **Lactulose, z-score** |  |  |  |  |  |  |  |  |  |  |  |  |  |  |
| at 3m of age |  | 0.87 | (0.15) |  | 0.89 | (0.14) |  | 0.034 | (0.037) |  | 0.054 | (0.049) |  |  |
| at 6m of age |  | 0.98 | (0.15) |  | 0.97 | (0.13) |  | 0.014 | (0.036) |  | -0.015 | (0.048) |  |  |
| 3, 6 month average |  | 0.857 | (0.19) |  | 0.884 | (0.17) |  | 0.059 | (0.048) |  | 0.028 | (0.064) |  |  |
| **Mannitol, z-score** |  |  |  |  |  |  |  |  |  |  |  |  |  |  |
| at 3m of age |  | 0.74 | (0.22) |  | 0.8 | (0.2) |  | 0.081 | (0.051) |  | 0.103 | (0.067) | ^††^ |  |
| at 6m of age |  | 1.08 | (0.2) |  | 0.69 | (0.18) | ^†^ | 0.039 | (0.047) |  | 0.163 | (0.063) | ^††^ |  |
| 3, 6 month average |  | 0.85 | (0.25) |  | 0.59 | (0.24) | ^†^ | 0.114 | (0.063) | ^*^ | 0.232 | (0.084) |  |  |
| **Lactulose-Mannitol Ratio, z-score** |  |  |  |  |  |  |  |  |  |  |  |  |  |  |
| at 3m of age |  | 1.19 | (0.22) |  | 1.2 | (0.21) |  | -0.032 | (0.052) |  | -0.08 | (0.068) | ^††^ |  |
| at 6m of age |  | 0.89 | (0.23) |  | 1.53 | (0.23) | ^*^ | -0.052 | (0.056) |  | -0.218 | (0.075) | ^††^ |  |
| 3, 6 month average |  | 0.98 | (0.24) |  | 1.67 | (0.27) | ^*^ | -0.049 | (0.067) |  | -0.25 | (0.089) |  |  |
| **Supplemental Table 3e. Base Model covariates plus biomarkers of Gut Function.** The collection, processing and calculation of gut function biomarkers has been described by Lee GO, McCormick BJJ, Seidman JC, et.al., (2017). | | | | | | | | | | | | | | |
| ††† p<0.001; †† p<0.01; † p<0.05; * p<0.10;  1 - Standard error of the log odds, not the OR | | | | | | | | | | | | | | |

**Statistical Model Description**

Here we describe the two multivariate models.

Model 1: Logit model for seroconversion failure. We specify this model by denoting $y_{ist}$ as a Bernoulli variable with the value 1 indicating failure and 0 indicating seroconversion, i.e., $y_{ist}=1$ for Log_2_(titre)<3; $y_{ist}=0$ for Log_2_(titre)≥3 for child *i* in site *s* at collection time *t*. The general form of the model is specified as:

$$logit\left[ \Pr\left( y_{ist}=1 \right)|u_{i} \right]=\beta_{0}+\beta_{1}x_{age,ist}+\boldsymbol{\beta}_{\boldsymbol{s}}\boldsymbol{X}_{\boldsymbol{s}}\boldsymbol{+}\beta_{2}x_{OPV,it}\boldsymbol{+}\sum_{p=1}^{P} x_{ip}\beta_{p}$$

where $x_{age,ist}$ denotes child’s age; $\boldsymbol{X}_{s}$is a categorical variable indexing site; $x_{OPV,it}$ is the number of OPV doses received; $x_{ip}$ represents additional variables evaluated; and $u_{i}$ is the random effect. Variables tested include vaccine history, enteropathogen detection, diarrhea, nutrient status, and socio-economic home environment, which were tested individually, adjusting for age at blood draw, site and OPV doses received. Variables were excluded from the final model selection if Akaike’s Information Criterion (AIC) did not indicate improved model fit. Selection of final variables proceeded sequentially starting with the variables indicating the lowest AIC and retaining variables that either improved model fit or whose Type III fixed effects test p-value was under 0.20.

Model 2: Linear mixed model for Log_2_(titer). Since both models are of the exponential family, they are specified nearly identically. Replacing $logit\left[ \Pr\left( y_{ist}=1 \right)|u_{i} \right]$ with Log_2_(titer) and adding in the random effect terms directly into the model, we write:

$${Log}_{2}\left[ \mathrm{titer} \right]=\beta_{0}+\beta_{1}x_{age,ist}+\boldsymbol{\beta}_{\boldsymbol{s}}\boldsymbol{X}_{\boldsymbol{s}}\boldsymbol{+}\beta_{2}x_{OPV,it}\boldsymbol{+}\sum_{p=1}^{P} x_{ip}\beta_{p}+u_{is}+e_{ist}$$

Both models will use AIC for model selection and both models tested additional variables individually, adjusting for age, site and OPV doses received.

**Model fit (Tables 2a, 2b)**

Here we compare the two models fit for each serotype.

For serotype 1, final models for seroconversion failure and Log_2_ titer both included variables for site, age at blood draw, OPV doses received (3, 4, or 5+), and age when solids were introduced (<2, 2-4, or >4 months of age). Seroconversion failure was also predicted by the non-diarrheal enteropathogen score at the blood draw, hemoglobin level, the assets component of WAMI and the Child Cleanliness score. In contrast, additional variables predicting Log_2_ serotype 1 titer included the non-diarrheal bacteria score, plasma transferretin receptor (TfR; <2.9, 2.9-8.3, or ≥8.3 µg/mL), and the overall WAMI index.

The final serotype 3 models both included site, age at blood draw, OPV doses received, non-diarrheal parasite (at blood draw) and bacteria score (at 12 weeks of age), age animal milk was introduced (<2, 2-4 and >4 months), and the overall WAMI index in the final models (Table 2b). Seroconversion failure for serotype 3 was also predicted by percent of days underweight (computed from monthly anthropometry scores and assumes last score carries forward). Categories of OPV doses received were different in each model.
